# Isolation of a Unique Monoterpene Diperoxy Dimer From *Ziziphora clinopodioides* subsp. *bungeana* Together With Triterpenes With Antidiabetic Properties

**DOI:** 10.1002/pca.3505

**Published:** 2025-01-08

**Authors:** Milan Malaník, Jakub Treml, Renata Kubínová, Gabriela Vávrová, Michal Oravec, Jaromír Marek, Karlygash Zhaparkulova, Liliya Ibragimova, Tolkyn Bekezhanova, Aigerim Karaubayeva, Zuriyadda Sakipova, Karel Šmejkal

**Affiliations:** ^1^ Department of Natural Drugs, Faculty of Pharmacy Masaryk University Brno Czech Republic; ^2^ Department of Molecular Pharmacy, Faculty of Pharmacy Masaryk University Brno Czech Republic; ^3^ Global Change Research Institute of the Czech Academy of Sciences Brno Czech Republic; ^4^ Core Facility Biomolecular Interactions and Crystallography, CEITEC MU Masaryk University Brno Czech Republic; ^5^ Department of Chemistry, Faculty of Science Masaryk University Brno Czech Republic; ^6^ School of Pharmacy Asfendiyarov Kazakh National Medical University Almaty Kazakhstan

**Keywords:** antidiabetic, GLUT4, pomolic acid, triterpenes, *Ziziphora*, α‐glucosidase

## Abstract

**Introduction:**

*Ziziphora clinopodioides* subsp. *bungeana* (Juz.) Rech.f. is used in traditional medicine for various purposes. Previous phytochemical studies focused on phenolic compounds, but triterpenoids were almost overlooked.

**Objective:**

The study focused on the isolation of compounds with dual antidiabetic activity from the aerial parts of *Z. clinopodioides* subsp. *bungeana*.

**Materials and Methods:**

Separation of CHCl_3_‐soluble fraction by silica gel column chromatography using different mobile phases and purification of compounds by semi‐preparative HPLC or preparative TLC. The structures of pure compounds were elucidated by 1D and 2D NMR experiments along with HRMS. Compound **1** was additionally identified by the single crystal X‐ray diffraction method. α‐Glucosidase inhibitory assay and GLUT4 expression and translocation in C2C12 myotubes were conducted to evaluate antidiabetic potential of isolated compounds.

**Results:**

This phytochemical study led to the isolation of 20 compounds, including a unique monoterpene diperoxy dimer (**1**). Compounds **7** and **9**–**11** displayed more potent α‐glucosidase inhibitory activity (IC_50_ 45.3–135.3 μM) than acarbose used as a positive control (IC_50_ 264.7 μM), while only pomolic acid (**5**) increased GLUT4 translocation in C2C12 myotubes in a significant manner.

**Conclusion:**

Extensive chromatographic separation led to the isolation and identification of a unique monoterpene diperoxy dimer (**1**) from aerial parts of *Z. clinopodioides* subsp. *bungeana*. Some triterpenes inhibited α‐glucosidase, another increased GLUT4 translocation. Although none of the isolated compounds demonstrated dual antidiabetic activity, selected triterpenes proved to be potent antidiabetic agents *in vitro*.

## 1 Introduction

*Ziziphora clinopodioides* subsp. *bungeana* (Juz.) Rech.f. (syn. *Z. bungeana* Juz.) is a typical member of the Lamiaceae family. It is spread throughout Central Asia, where it grows on gravelly hillsides or sandy areas up to 1100 m above sea level [1]. The plant is used in the traditional medicine of many Asian countries to treat infectious diseases, especially of the respiratory system. In Uyghur medicine, *Z. bungeana* is used to alleviate symptoms associated with cardiovascular diseases [1]. On the other hand, the folk medicine of Iran describes the use of *Z. clinopodioides* subsp. *bungeana* in the form of infusions to reduce fever, for stomach aches, or for antiseptic, sedative, and carminative purposes [2].

Unfortunately, *Z. clinopodioides* subsp. *bungeana* is still overlooked for some reason, and there is only limited information about its phytochemical profile. As for other *Ziziphora* species, the essential oil obtained from *Z. clinopodioides* subsp. *bungeana* is rich in pulegone [3], supporting its use in folk medicine against infectious diseases. A recent review comprehensively delineates the content compounds in the most common *Ziziphora* species [1] and points out that there are almost no publications dealing with the non‐volatile constituents of *Z. clinopodioides* subsp. *bungeana*. More recently, β‐sitosterol, betulin, and two rare oleanane derivatives have been isolated from the aerial parts of the plant [4], and He et al. have detected the presence especially of flavonoid glycosides in different parts of *Z. bungeana* [5]. Very recently, Zhaparkulova et al. characterized the composition of extracts of different polarities using HPLC‐ESI‐QTOF‐MS/MS, revealing especially the presence of various phenolics [6]. Very similar content compounds have been described in a study dealing with the cardiotropic activity of extracts of the aerial parts of *Z. clinopodioides* subsp. *bungeana* [7]. As can be seen, the attention of scientists is focused mainly on phenolic compounds, although *Z. clinopodioides* subsp. *bungeana* seems to be also rich in triterpenoids. Therefore, this study aims to isolate and structurally characterize further compounds to broaden the knowledge of the phytochemical profile of *Z. clinopodioides* subsp. *bungeana* and to support the use of this underestimated plant.

Although it is assumed that especially flavonoids are responsible for the use of *Z. bungeana* in folk medicine and the vast majority of phytochemical studies of *Ziziphora* species are aimed accordingly [1, 8, 9], our initial study revealed that extract of the aerial parts of *Z. bungeana* is rich in compounds lacking chromophores, mostly triterpenoids [10]. Triterpenes are natural compounds widespread in the plant kingdom and are known for their antidiabetic properties [11]. The inhibition of α‐glucosidase and α‐amylase is the main mechanism of action of triterpenes, but other diabetes‐related enzymes like protein tyrosine phosphatase 1B and aldose reductase can be affected by triterpenes as well [11]. Moreover, some triterpenes have been described as effective insulin‐mimetic agents leading to increased GLUT4 translocation that indicates their diverse mechanism of antidiabetic activity [12]. Taken together with previously described rare triterpenoids [4], we found *Z. clinopodioides* subsp. *bungeana* to be an attractive source for the isolation of structurally diverse triterpenes with potentially even more distinct and multiple mechanisms of antidiabetic action.

An extract of the aerial parts of *Z. clinopodioides* subsp. *bungeana* was subjected to extensive chromatographic separation leading to the isolation and identification of 20 structurally diverse compounds, including the unique monoterpene diperoxy dimer (**1**) isolated from natural material for the first time. The α‐glucosidase inhibitory activity and the effect on GLUT4 translocation of selected compounds have been evaluated to find triterpenes with potential dual antidiabetic activity.

## 2 Materials and Methods

### 2.1 General Experimental Procedures

**FIGURE 1:**
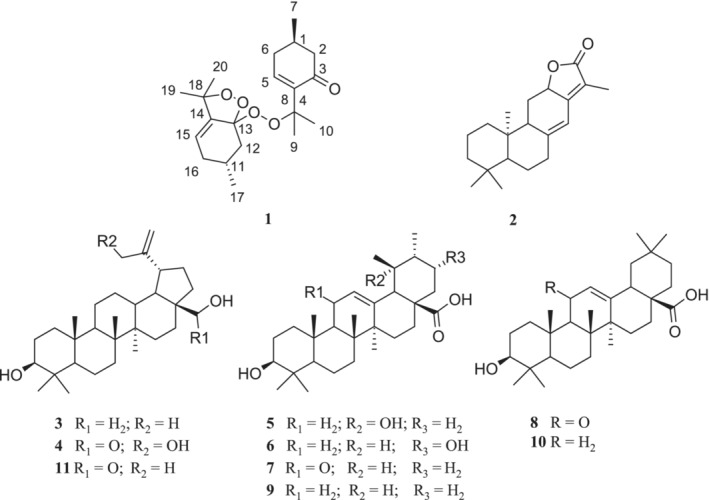
Structures of the compounds **1**–**11** isolated from aerial parts of *Z. clinopodioides* subsp. *bungeana*.

**FIGURE 2:**
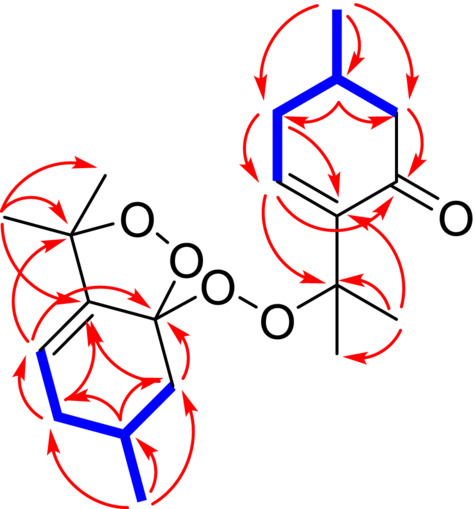
Key ^1^H‐^1^H COSY (blue bold lines) and HMBC (red arrows) correlations of compound **1.**

IR spectra were determined by the ATR method on a Nicolet Impact 400D FT‐IR spectrophotometer (Nicolet, USA). NMR (1D and 2D) spectra were obtained using a JEOL ECZR 400‐MHz spectrometer (Jeol, Japan). HRMS analysis was performed using an LTQ Orbitrap XL‐high resolution mass spectrometer equipped with an HESI II (Heated electrospray ionization) source. Samples were analyzed twice on HPLC‐HRMS with positive and negative polarity of the MS‐Orbitrap. Column chromatography (CC) was carried out using silica gel with a particle size of 0.040–0.063 mm (Merck, USA). For preparative purposes, TLC plates (Uniplate, silica gel G, 20 × 20 cm, 500 μm; Analtech, USA) were used. Analytical HPLC analysis of compounds absorbing UV radiation was measured on an Agilent 1100 instrument (Agilent, Germany) equipped with a DAD using an Ascentis Express RP‐Amide analytical column (100 mm × 2.1 mm, particle size 2.7 μm) (Supelco, USA). For the analysis of compounds lacking chromophores, a Dionex UltiMate 3000 liquid chromatograph (Thermo Scientific, Germany) equipped with an evaporative light scattering detector (ELSD) was used. Semipreparative HPLC was performed using an Ascentis RP‐Amide column (250 mm × 10 mm, particle size 5 μm) with an HPLC system Young Lin 9100 (Young Lin, South Korea) with PDA or a Dionex UltiMate 3000 liquid chromatograph equipped with a UV detector or ELSD (Varian, UK). HPLC solvents (MeOH and MeCN) were purchased from VWR International, France, and other analytical grade solvents were obtained from Lach‐Ner, Czech Republic. Marvin 24.1.2, Chemaxon (https://www.chemaxon.com), was used to draw the chemical structures of the isolated compounds (Figure 1), and ChemDraw 23.1.1, Revvity Signals (https://revvitysignals.com), was used to draw the key ^1^H‐^1^H COSY and HMBC correlations of compound **1** (Figure 2).

### 2.2 Plant Material

The aerial parts of *Z. clinopodioides* subsp. *bungeana* (Juz.) Rech.f. were collected in summer 2014 in the flowering stage at the foothills of the Dzungarian Alatau, Republic of Kazakhstan, and identified by Dr. N. G. Gemejiyeva (Institute of Botany and Phytointroduction, Science Committee‐Ministry of Education and Science of the Republic of Kazakhstan). A voucher specimen (no. 01‐04/257) has been deposited in the herbarium of the Institute of Botany and Phytointroduction, Almaty, Republic of Kazakhstan.

### 2.3 Extraction and Isolation Procedures

The air‐dried aerial parts of *Z. clinopodioides* subsp. *bungeana* (393 g) were extracted with 96% EtOH (3 × 24 h). The solvent was removed using a rotavapor to obtain 42.6 g of crude extract that was partitioned consecutively with *n*‐hexane, CHCl_3_, and EtOAc. The CHCl_3_‐soluble extract (9.37 g) was further processed by silica gel CC using different mobile phases and several columns with different proportions. Final purifications were achieved using semi‐preparative HPLC or preparative TLC. Schema of the separation procedure and the isolation of the pure compounds can be found in the Supporting Information (Figure S10).

#### 2.3.1 Ziziphora Diperoxy Dimer **1**

Amorphous colorless substance; UV (MeOH) λ_max_ (log ε) 231 (4.14) nm; IR (ATR) *v*_max_ 2936, 2819, 2160, 1652, 1456, 1376, 1361, 1259, 1195, 1022, 1016, 928, 858, 850, 845, 833, 820 cm^−1^; ^1^H and ^13^C NMR data; see Table 1; HRESIMS *m/z* 373.1995 [M + Na]^+^ (calcd for C_20_H_30_O_5_Na, 373.1991). Complete spectroscopic data can be found in the Supporting Information (Figure S1–S9).

### 2.4 X‐ray Crystallographic Analysis of Compound **1**

A single crystal of **1** was obtained by vapor diffusion of MeOH at reduced temperature (4°C–8°C). X‐ray diffraction data were collected at 120.00 (10) K with a Rigaku Oxford Diffraction Synergy Custom system and a Rigaku HyPix‐6000HE detector using Mo Kα radiation (*λ* = 0.710 73 Å) generated by a Rigaku MicroMax‐007 HF DW. The data collection, reduction, and absorption corrections were performed using CrysAlisPro (version 1.171.41.68a). The crystal structure was solved and refined using SHELXT [13] and SHELXL [14]. To achieve higher accuracy, especially for the positions of the hydrogen atoms, the final refinement steps were performed using the NoSpherA2 function [15] in Olex2 [16, 17], where ORCA 5.0 (method R2SCAN and basis set cc‐pVTZ) [18] was used to generate nonspherical atomic form factors. Crystal data and final agreement parameters can be found in the Supporting Information. The Crystallographic Information File for **1** has been deposited in the Cambridge Crystallographic Data Centre (CCDC deposition number 2380893), from which it can be obtained free of charge using https://www.ccdc.cam.ac.uk/structures/.

### 2.5 Inhibition of α‐Glucosidase

The inhibition of α‐glucosidase was performed according to the standard method as described previously [19, 20] with a slight modification: 160 μL of 0.1‐M phosphate buffer (Na_2_HPO_4_ 2H_2_O, pH 7.0), 20 μL of enzyme solution (1 U/mL), and 20 μL of the test compound were mixed. After incubation (15 min, 37°C), the reaction was initiated by adding 20 μL of 2.5‐mM *p*‐nitrophenyl‐α‐D‐glucopyranoside. After an additional 15 min at 37°C, the reaction was stopped with 80 μL of 0.2‐M Na_2_CO_3_. The released *p*‐nitrophenol was quantified spectrophotometrically at λ 405 nm using a microplate reader. One set of mixtures prepared with an equivalent volume of methanol instead of the test samples was used as a control. Another set of mixtures prepared with an equivalent volume of phosphate buffer instead of the enzyme was used as a blank. The inhibition of the enzyme (%) was calculated according to the formula: [1 − (Abs_sample_ − Abs_control_) /Abs_blank_] × 100. Acarbose was used as the reference compound.

### 2.6 Cell Culture

The mouse myoblast C2C12 cell line was purchased from the European Collection of Authenticated Cell Cultures (Salisbury, UK). The C2C12 cell line was cultured in DMEM High Glucose medium (Biosera, Nuaille, France), supplemented with antibiotics (100 U/mL penicillin and 100 mg/mL streptomycin (Biosera), and 10% fetal bovine serum (FBS) (HyClone, Logan, UT, USA). The cells were kept in an incubator at 37°C in a water‐saturated atmosphere of air containing 5% CO_2_. The cells were detached using a solution of trypsin EDTA 1 × in PBS (Biosera) and passaged approximately twice a week.

### 2.7 GLUT4 Translocation Experiment and Isolation of Plasma Membrane Fraction

The C2C12 cell line was incubated in containers with a surface area of 75 cm^2^ (tissue‐culture treated) to 100% confluence in full serum DMEM High Glucose medium. Then the culture medium was changed to DMEM High Glucose medium, supplemented with antibiotics, and 2% horse serum (Biosera) to start the differentiation of myoblasts into myocytes. The medium was renewed every 2 days, and the formation of myocytes was observed using a microscope. After 6 days of differentiation, the culture medium was changed to DMEM High Glucose serum‐free medium, and the test compounds were added at a non‐toxic concentration of 5 μM dissolved in DMSO. The effect of the test compounds on the viability of C2C12 myocytes was determined using a WST‐1 kit (Roche, Basel, Switzerland), according to the manufacturer's manual. The final concentration of DMSO in the medium was 0.1% (v/v). After 24 h of incubation, the cells were collected and lysed with 135 μL of buffer A with 0.1% (v/v) Nonidet™ P 40 Substitute (Sigma Aldrich). The buffer A composed of 50‐mM Tris–HCl (Serva, Germany), pH 8.0; 0.5 DTT (Carl Roth, Germany); 10‐mM NaF (Penta Chemicals, Czech Republic); 1‐mM Na_3_VO_4_ (Sigma Aldrich); complete™ Protease Inhibitor Cocktail (Roche). Experiments for each test compound and the solvent control (DMSO) were done in quadruplicate. The isolation of plasma membrane proteins was performed according to the procedure described by Yamamoto et al. [21] with slight modifications. Mainly, the relative centrifugation force for collecting the plasma membrane proteins was increased from 750 × *g* to 14,000 × *g*.

### 2.8 Polyacrylamide Gel Electrophoresis (PAGE) and Western Blot

The effects of the test compounds on the protein expression of GLUT4 in the total cellular fraction and on GLUT4 translocation to the plasma membrane fraction were measured using PAGE and Western blot with specific antibodies. We employed the method described by Treml et al. [22] with slight modifications to separate proteins in fractions from the previous experiment. To detect the proteins of our interest [GLUT4, β‐actin, and Na^+^/K^+^ ATPase (ATPA1)], we used rabbit anti‐GLUT4 1:2000 (ThermoFisher Scientific; Waltham, MA, USA; product no. PA5–23052), mouse anti‐ATP1A1 1:2000 (ThermoFisher Scientific; product no. MA3–928), or mouse anti‐β‐actin 1:5000 (Abcam; product no. ab8226).

To quantify the effects of the test compounds on GLUT4 expression and translocation, the optical densities (OD) of the corresponding bands shown by Western blot analysis were calculated and expressed as a ratio of OD_GLUT4_ / OD_β‐actin_ for GLUT4 expression in whole‐cell lysates. Similarly, GLUT4 translocation was expressed as a ratio of OD_GLUT4_/OD_ATPA1_ in the plasma membrane fraction, since ATPA1 is an enzyme present in the plasma membrane.

### 2.9 Statistical Analysis

Statistical analysis was carried out using IBM SPSS Statistics for Windows, software version 26.0 (Armonk, NY, USA). The data were graphed as the mean ± SEM. Comparisons between groups were made using a Mann–Whitney *U* test.

## 3 Results and Discussion

### 3.1 Identification of 20 Isolated Compounds

Separation procedure led to the isolation of 20 chemically diverse compounds which were structurally characterized on the basis of extensive NMR and MS analysis. The structure of compound **1** was finally elucidated using X‐ray diffraction analysis which unambiguously identified **1** as a unique monoterpene diperoxy dimer. The existence of this type of compound is reported here for the first time. Further 10 known compounds (Figure 1) were selected for evaluation of their antidiabetic activity and, based on the comparison of their spectroscopic data with those in the literature, were identified as follows: jolkinolide E (**2**) [23], betulin (**3**) [24], 30‐hydroxybetulinic acid (syn. messagenic acid D) (**4**) [25], pomolic acid (**5**) [26], 21α‐hydroxyursolic acid (**6**) [27], 11‐oxoursolic acid (**7**) [28], 11‐oxooleanolic acid (**8**) [28], ursolic acid (**9**) [28], oleanolic acid (**10**) [28], and betulinic acid (**11**) [29]. Although the vast majority of the other identified compounds were also isolated from *Z. clinopodioides* subsp. *bungeana* for the first time, the compounds loliolide, piceol, herniarin, eupatorin, chrysin, genkwanin, apigenin, cinnamic acid, and vanillic acid were not included in the biological tests because their biological activities have already been reported in numerous studies.

**FIGURE 3:**
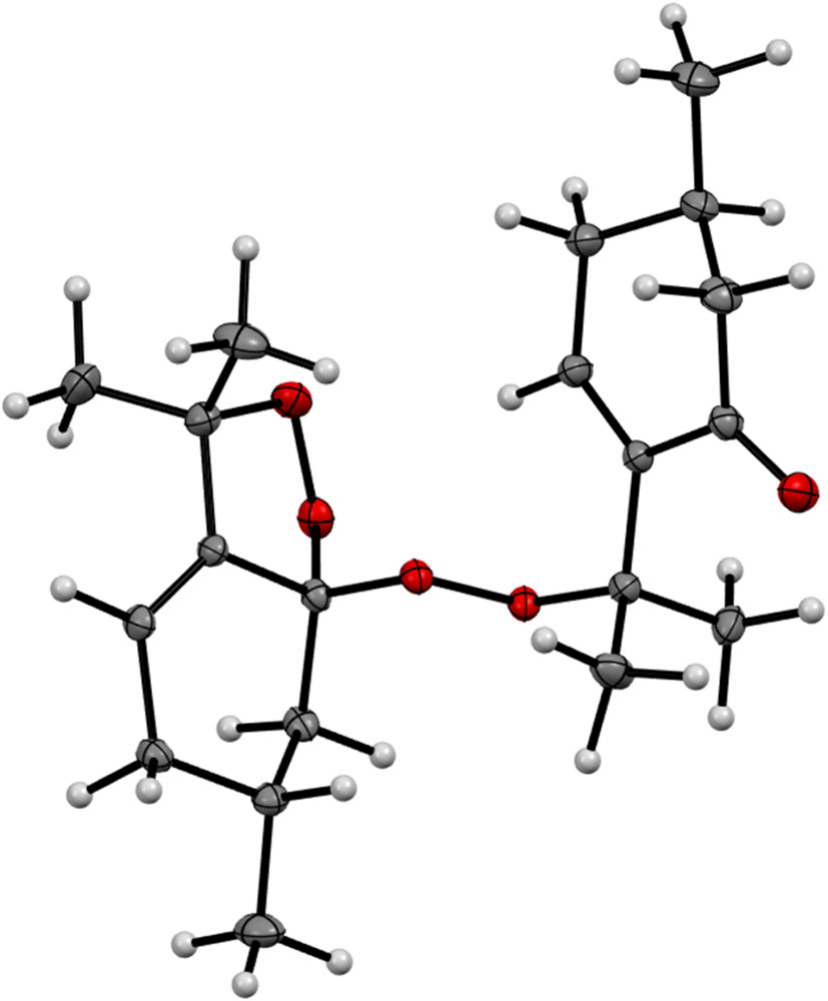
ORTEP diagram of compound **1.**

Compound **1** was obtained as an amorphous colorless powder. Its molecular formula was established as C_20_H_30_O_5_ based on the HRESIMS ion at *m/z* 373.1995 [M + Na]^+^ (calcd for C_20_H_30_O_5_Na, 373.1991). The ^1^H NMR data (Table 1) revealed the presence of two secondary methyl groups [δ_H_ 1.01 and 1.07 (each 3H, d)] and four tertiary methyl groups [δ_H_ 1.31–1.48 (each 3H, s)]. In general, the aliphatic region was overcrowded with many signals corresponding to methines and methylenes [δ_H_ 1.05–2.63 (each 1H, m)]. Furthermore, only two olefinic protons [δ_H_ 5.62 (1H, t, *J* = 3.6 Hz) and δ_H_ 7.29 (1H, dd, *J* = 5.6, 2.8 Hz)] could be observed in the ^1^H NMR spectrum. A detailed evaluation of the ^1^H‐^1^H COSY, HSQC, and HMBC data (Figure 2) revealed the presence of two separate ten‐carbon units—one is a pulegone‐like unit and the other containing a *p*‐meth‐3‐ene skeleton. However, both units must be somehow connected. Based on the established molecular formula, compound **1** contained five oxygen atoms, and the presence of only one carbonyl group (δ_C_ 198.9) has been revealed so far. Therefore, the presence of a peroxy linkage between the above mentioned monoterpene units was proposed in accordance with recently published data for *p*‐menthene‐type monoterpene peroxy dimers [30]. However, two oxygen atoms still remained, and based on the evaluation of ^13^C NMR data, only three carbon atoms (δ_C_ 81.9, 82.4, and 107.9) were considered to be possibly connected by peroxy linkages. Unfortunately, it was not possible to determine the positions of the possible peroxy linkages and to finally elucidate the structure solely by NMR analysis. Thus, it was necessary to carry out an X‐ray diffraction analysis (Table S1) that unambiguously identified **1** as a unique monoterpene diperoxy dimer (Figure 3) that was named ziziphora diperoxy dimer.

**TABLE 1:** ^1^H and ^13^C NMR chemical shifts (*δ* in ppm) of compound **1** in CDCl_3_.

No.	δ_C_, type	δ_H_ (*J* in Hz)
1	30.3, CH	2.17, m
2	48.1, CH_2_	2.10, m
		2.43, m
3	198.9, C	
4	141.3, C	
5	145.6, CH	7.29, dd (5.6, 2.8)
6	34.4, CH_2_	2.02, m 2.49, m
7	21.1, CH_3_	1.01, d (6.2)
8	82.4, C	
9	25.0, CH_3_	1.48, s
10	25.8, CH_3_	1.45, s
11	24.9, CH	2.04, m
12	33.7, CH_2_	1.05, overlapped 2.63, dd (12.6, 3.3)
13	107.9, C	
14	147.0, C	
15	123.4, CH	5.62, t (3.6)
16	34.1, CH_2_	1.66, ddd (13.7, 10.3, 3.3)
		2.34, ddd (9.6, 5.8, 3.6)
17	21.2, CH_3_	1.07, d (6.8)
18	81.9, C	
19	28.3, CH_3_	1.36, s
20	25.5, CH_3_	1.31, s

### 3.2 Inhibition of α‐Glucosidase

To investigate the α‐glucosidase inhibitory potential, compounds (**1**–**11**) were subjected to testing using a microplate reader. Although the α‐glucosidase inhibition of some of the selected triterpenes has been described previously several times, their values of IC_50_ in the literature differ dramatically. Therefore, we tested all compounds (both tested and untested previously) at the same concentration (200 μM) to compare the results easily and to determine the structure–activity relationship. Acarbose was used as a positive control. The results are summarized in Table 2 and expressed as the mean ± SEM for three independent experiments measured in triplicate.

**TABLE 2:** Inhibition of α‐glucosidase and IC_50_ values of the most active constituents.

Compounds	% of inhibition (200 μM)	IC_50_ values (μM)
Ziziphora diperoxy dimer (**1**)	6.0 ± 1.9	> 200
Jolkinolide E (**2**)	25.0 ± 1.4	> 200
Betulin (**3**)	24.0 ± 6.0	> 200
30‐Hydroxybetulinic acid (**4**)	34.0 ± 4.9	> 200
Pomolic acid (**5**)	41.9 ± 4.1	> 200
21α‐Hydroxyursolic acid (**6**)	26.8 ± 2.1	> 200
11‐Oxoursolic acid (**7**)	56.0 ± 5.7	162.7 ± 15.4
11‐Oxooleanolic acid (**8**)	49.9 ± 3.3	> 200
Ursolic acid (**9**)	65.7 ± 4.1	80.9 ± 2.3
Oleanolic acid (**10**)	67.5 ± 1.4	135.3 ± 9.0
Betulinic acid (**11**)	64.4 ± 2.0	45.3 ± 1.0
Acarbose	53.3 ± 7.6	264.7 ± 25.8

The results indicate that triterpenic acids most commonly occurring in nature [ursolic acid (**9**), oleanolic acid (**10**), betulinic acid (**11**)] exerted the most potent α‐glucosidase inhibitory activity with IC_50_ values ranging from 45.3 to 135.3 μM. All of these three triterpenic acids together with 11‐oxoursolic acid (**7**) were more active than acarbose (IC_50_ = 264.7 ± 25.8 μM). Pentacyclic triterpenes are known for their α‐glucosidase inhibitory properties [11], and the results from our study confirm this. Betulinic acid (**11**) was the most active compound, which is consistent with the literature where betulinic acid (**11**) is described as a strong α‐glucosidase inhibitor that binds to the active cavity of α‐glucosidase and thus reduces the activity of the enzyme [31]. Furthermore, ursolic acid (**9**) and oleanolic acid (**10**) are both of great significance regarding α‐glucosidase inhibitory activity as they have been designated as allosteric inhibitors of this enzyme [32]. The combination of ursolic acid (**9**) and oleanolic acid (**10**) displayed even more significant synergistic inhibition of α‐glucosidase [32], which is a very beneficial finding, especially in connection with the challenging separation of these regioisomers.

Initially, we assumed that hydroxy substitution could improve α‐glucosidase inhibitory activity as this has been described, for example, for corosolic acid, a 2α‐hydroxy derivative of ursolic acid [33, 34]. Unfortunately, none of the other compounds tested in this assay reached at least 50% inhibition of the enzyme, except for 11‐oxoursolic acid (**7**). This finding is surprising in view of the fact that glycyrrhetic acid differing from 11‐oxooleanolic acid (**8**) only in the position of the carboxy group has been described previously as inactive [35]. Therefore, we conclude that the position of the carboxy group (C‐28) plays a crucial role in the inhibition of α‐glucosidase as do the positions of further hydroxy groups. The literature reports that 2α‐hydroxy substitution significantly increased the activity [33, 34], whereas for the compounds tested in our study, hydroxy substitution at any other position decreased the inhibition of α‐glucosidase.

### 3.3 GLUT4 Expression and Translocation

**FIGURE 4:**
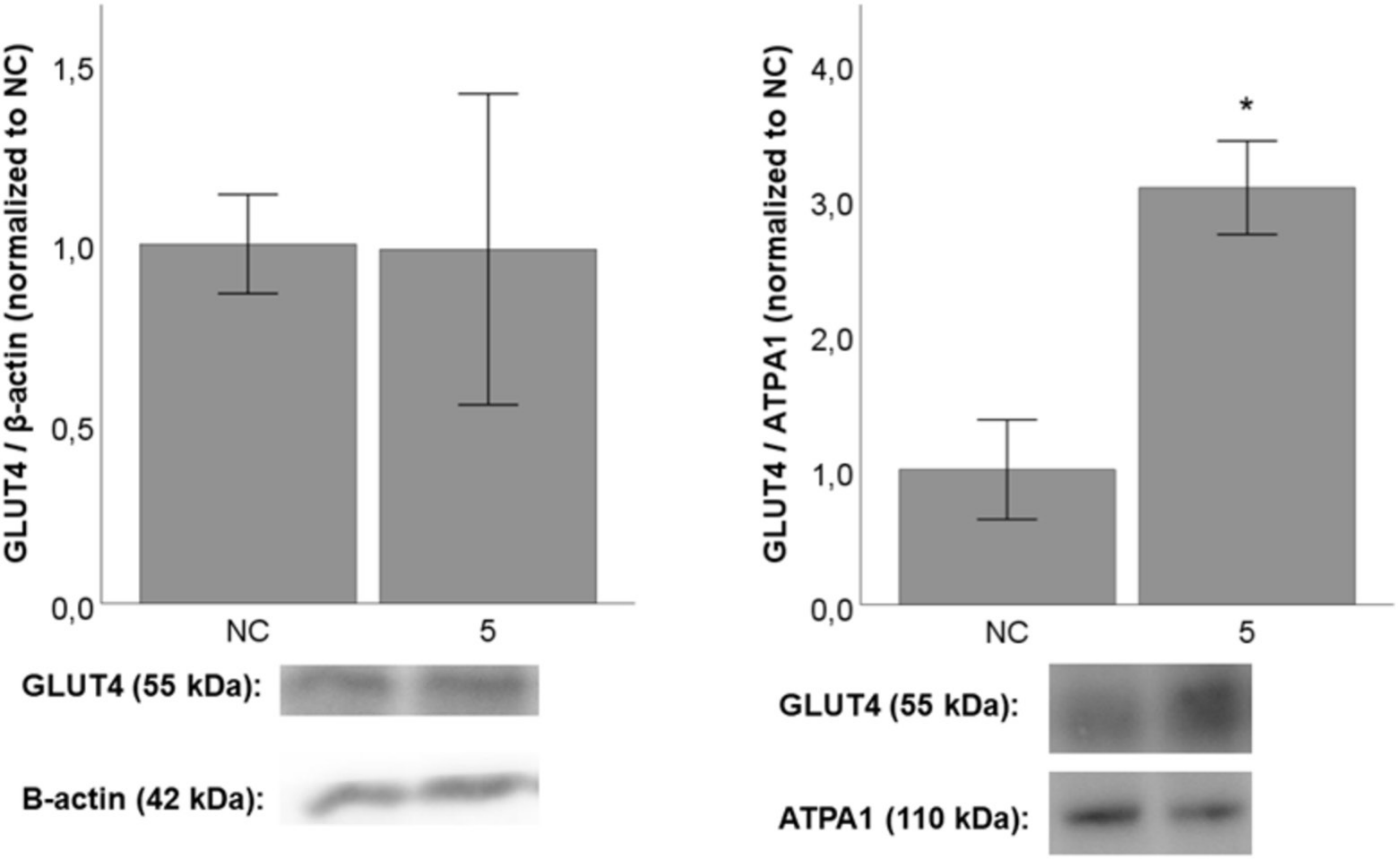
The effect of pomolic acid (**5**) at a concentration of 5 μM on GLUT4 expression and translocation in C2C12 myotubes. DMSO used as solvent served as the negative control (NC). The results are expressed as the mean ± SEM for four independent experiments and are statistically compared to NC (* *p* < 0.05).

To elucidate the effects of the test compounds on the expression and translocation of GLUT4, the test compounds were incubated for 24 h with C2C12 differentiated myotubes at a non‐toxic concentration of 5 μM (data for toxicity studies not shown). None of the test compounds showed a significant effect on either GLUT4 expression or translocation (data not shown), apart from pomolic acid (**5**) which was able to increase GLUT4 translocation in C2C12 myotubes (marked by tripling the GLUT4/ATPA1 ratio) in a significant manner (*p* < 0.05) as shown in Figure 4.

Our results are in accordance with the findings of Lee and Thuong who showed a positive effect of pomolic acid (**5**) on 2‐deoxy‐D‐[3H]‐glucose uptake in L6 myotube cells with uptake increased by 1.6‐fold [36]. Moreover, Thuong et al. reported that pomolic acid (**5**) inhibits the protein tyrosine phosphatase 1B (PTP1B)—an enzyme down‐regulating insulin pathway, with IC_50_ = 3.9 ± 0.8 μM [37]. On the other hand, two test compounds with no activity in our experiment showed PTP1B inhibitory activity in another study in a similar or even better manner:ursolic acid (**9**) with IC_50_ = 3.1 ± 0.2 μM [37] and betulinic acid (**11**) with IC_50_ = 0.7 ± 0.03 μg/mL (= 1.53 μM) [38]. Moreover, Jung et al. reported that ursolic acid (**9**) potentiated insulin mediated tyrosine phosphorylation of the insulin receptor β‐subunit, the phosphorylation of Akt, the glycogen synthase kinase‐3β, and enhanced the translocation of GLUT4 in 3 T3‐L1 adipocytes [12]. Similarly, Lee and Thuong reported that ursolic acid (**9**) was active in their study (1.84‐fold increase) [36]. The discrepancy between our results and the reported ones might be explained by differences in methodologies. Also, the inhibition of PTP1B enzymes seems not to be always directly connected to increased GLUT4 translocation and glucose uptake.

## Supporting information


**Table S1.** Crystal data and structure refinement for **1** (CCDC 2380893).
**Figure S1.** UV spectrum of compound **1**.
**Figure S2.** IR spectrum of compound **1**.
**Figure S3.** HRESIMS spectrum of compound **1** in positive mode.
**Figure S4.** ^1^H NMR spectrum of compound **1** in CDCl_3_.
**Figure S5.** ^13^C NMR spectrum of compound **1** in CDCl_3_.
**Figure S6.** HSQC spectrum of compound **1** in CDCl_3_.
**Figure S7.** HMBC spectrum of compound **1** in CDCl_3_.
**Figure S8.** ^1^H‐^1^H COSY spectrum of compound **1** in CDCl_3_.
**Figure S9.** NOESY spectrum of compound **1** in CDCl_3_.
**Figure S10.** Schema of the separation procedure.

## Data Availability

The authors confirm that the data supporting the findings of this study are available within the article and in the Supporting information. Further data are available from the corresponding author upon reasonable request.
