# Supplementary material for: Isolation of a Unique Monoterpene Diperoxy Dimer From Ziziphora clinopodioides subsp. bungeana Together With Triterpenes With Antidiabetic Properties
Source: Phytochem Anal. 2025 Jan 8;36(4):1223–30. doi: 10.1002/pca.3505 (PMC12129716; doi:10.1002/pca.3505)
Supplement: Supplementary file 1 — Table S1. Crystal data and structure refinement for 1 (CCDC 2380893). Figure S1. UV spectrum of compound 1. Figure S2. IR spectrum of compound 1. Figure S3. HRESIMS spectrum of compound 1 in positive mode. Figure S4. 1H NMR spectrum of compound 1 in CDCl3. Figure S5. 13C NMR spectrum of compound 1 in CDCl3. Figure S6. HSQC spectrum of compound 1 in CDCl3. Figure S7. HMBC spectrum of compound 1 in CDCl3. Figure S8. 1H‐1H COSY spectrum of compound 1 in CDCl3. Figure S9. NOESY spectrum of compound 1 in CDCl3. Figure S10. Schema of the separation procedure. [file PCA-36-1223-s001.docx]

Isolation of a unique monoterpene diperoxy dimer from *Ziziphora clinopodioides* subsp. *bungeana* together with triterpenes with antidiabetic properties

Milan Malaník^1,^*, Jakub Treml^2^, Renata Kubínová^1^, Gabriela Vávrová^1^, Michal Oravec^3^, Jaromír Marek^4,5^, Karlygash Zhaparkulova^6^, Liliya Ibragimova^6^, Tolkyn Bekezhanova^6^, Aigerim Karaubayeva^6^, Zuriyadda Sakipova^6^, and Karel Šmejkal^1^

^1^Department of Natural Drugs, Faculty of Pharmacy, Masaryk University, Palackého třída 1946/1, 61200 Brno, Czech Republic

^2^Department of Molecular Pharmacy, Faculty of Pharmacy, Masaryk University, Palackého třída 1946/1, 61200 Brno, Czech Republic

^3^Global Change Research Institute of the Czech Academy of Sciences, Bělidla 986/4a, 60300 Brno, Czech Republic

^4^Core Facility Biomolecular Interactions and Crystallography, CEITEC MU, Masaryk University, Kamenice 5, 62500 Brno, Czech Republic

^5^Department of Chemistry, Faculty of Science, Masaryk University, Kamenice 5, 62500 Brno, Czech Republic

^6^School of Pharmacy, Asfendiyarov Kazakh National Medical University, Tole-bi 94, 050012 Almaty, Kazakhstan

*Corresponding author.

*E-mail address*: malanikm@pharm.muni.cz (M. Malaník).

**Table of Contents**

**Table S1**. Crystal data and structure refinement for **1** (CCDC 2380893)

**Figure S1**. UV spectrum of compound **1**

**Figure S2**. IR spectrum of compound **1**

**Figure S3**. HRESIMS spectrum of compound **1** in positive mode

**Figure S4**. ^1^H NMR spectrum of compound **1** in CDCl_3_

**Figure S5**. ^13^C NMR spectrum of compound **1** in CDCl_3_

**Figure S6**. HSQC spectrum of compound **1** in CDCl_3_

**Figure S7**. HMBC spectrum of compound **1** in CDCl_3_

**Figure S8**. ^1^H-^1^H COSY spectrum of compound **1** in CDCl_3_

**Figure S9**. NOESY spectrum of compound **1** in CDCl_3_

**Figure S10**. Schema of the separation procedure

**Table S1**. Crystal data and structure refinement for **1** (CCDC 2380893)

Identification code 1

Empirical formula C20 H30 O5

Formula weight 350.458

Temperature 120.00(10) K

Wavelength 0.71073 Å

Crystal system, space group Monoclinic,  *P* 1 2_1_ 1

Unit cell dimensions a = 10.38375(18) Å α = 90°

b = 6.60943(10) Å β = 92.4561(16)°

c = 14.4055(3) Å γ = 90°

Volume 987.75(3) Å^3^

Z, Calculated density 2, 1.178 Mg . m^-3^

Absorption coefficient 0.083 mm^-1^

F(000) 380.253

Crystal size 0.22 x 0.12 x 0.08 mm

Theta range for data collection 1.96° to 31.11°

Limiting indices -14 <= h <= 12, -8 <= k <= 9, -18 <= l <= 19

Reflections collected / unique 13491 / 4958 [ R_int_ = 0.0132 ]

Completeness to theta = 25.2417 99.95 %

Absorption correction multi-scan

Max. and min. transmission 1.00000 and 0.74490

Refinement method Full-matrix least-squares on F^2^

Data / restraints / parameters 4958 / 1 / 496

Goodness-of-fit on F^2^ 1.2100

Final R indices [I>2σ(I)] R1 = 0.0131, wR2 = 0.0263

R indices (all data) R1 = 0.0138, wR2 = 0.0264

Absolute structure parameter 0.07(15)

Largest diff. peak and hole 0.0674 and -0.0650 e . Å^-3^

**Figure S1**. UV spectrum of compound **1**

**Figure S2**. IR spectrum of compound **1**

**Figure S3**. HRESIMS spectrum of compound **1** in positive mode

**Figure S4**. ^1^H NMR spectrum of compound **1** in CDCl_3_

**Figure S5**. ^13^C NMR spectrum of compound **1** in CDCl_3_

**Figure S6**. HSQC spectrum of compound **1** in CDCl_3_

**Figure S7**. HMBC spectrum of compound **1** in CDCl_3_

**Figure S8**. ^1^H-^1^H COSY spectrum of compound **1** in CDCl_3_

**Figure S9**. NOESY spectrum of compound **1** in CDCl_3_

**Figure S10**. Schema of the separation procedure

* Subfraction ZB-FII-21-22 (1.591 g) contains especially a mixture of ursolic acid (**9**) and oleanolic acid (**10**). Pure compounds were isolated from 50 mg of this subfraction.
